# Future support on evidence-informed priority setting and situational analysis of the potential role of Health Technology Assessment in Africa to support future pandemic preparedness and response: protocol for a scoping review

**DOI:** 10.1186/s13643-024-02610-4

**Published:** 2024-07-26

**Authors:** Benjamin S. C. Uzochukwu, Chinyere Okeke, Francis Ruiz, Sergio Torres-Rueda, Joseph Kazibwe, Adaora Uzochukwu, Anna Vassall

**Affiliations:** 1 Department of Compreparedness and Responsemunity Medicine, University of Nigeria Nsukka, Enugu Campus, Enugu, Nigeria; 2 Department of Global Health & Development, Faculty of Public Health and Policy, London School of Hygiene and Tropical Medicine, Keppel St, London, WC1E 7HT UK; 3 Department of Management, Faculty of Business Administration, University of Nigeria Nsukka, Enugu Campus, Enugu, Nigeria

## Abstract

**Background:**

The COVID-19 pandemic has highlighted the importance of evidence-informed priority setting and situational analysis in pandemic preparedness and response. Health Technology Assessment (HTA) has been identified as an essential tool for evidence-informed decision-making in healthcare. However, the potential role of HTA in pandemic preparedness and response in Africa has yet to be explored. The objective of this scoping review is to ascertain the current understanding of the possible role of HTA in Africa to support future pandemic preparedness and response.

**Methods:**

We will conduct a scoping review of literature published between 2010 and 2024. Electronic databases like Embase, PubMed, Scopus, Web of Science, and Google Scholar will be utilized to perform the search. We will also search grey literature sources such as websites of relevant organizations and government agencies. The search will only include studies that were conducted in the English language. Two reviewers will evaluate the titles and abstracts of the publications independently to determine their eligibility using Covidence. Full-text articles will be reviewed for eligibility and data extraction. The data will be extracted using a standardized form. The extracted data will include information on the study design, objectives, methods, findings, and conclusions. The thematic analysis approach will guide the data analysis. Themes and sub-themes will be identified and reported. The review will be reported following the Preferred Reporting Items for Systematic Reviews and Meta-Analyses Extension for Scoping Reviews (PRISMA-ScR) guidelines.

**Discussion:**

This scoping review will identify the existing knowledge on the potential role of HTA in Africa to support future pandemic preparedness and response. The findings will aid in identifying deficiencies in knowledge and provide valuable insights for future study. Additionally, they will inform policy-makers and other stakeholders about the potential contribution of the Health Technology Assessment (HTA) in enhancing Africa’s readiness and response to pandemics.

## Background

Emerging infections can carry substantial health and economic consequences, particularly for the most vulnerable countries and populations [1]. The COVID-19 pandemic, and previous epidemics including swine flu (H1N1), Ebola, Polio, Zika, and monkeypox have been officially designated as public health emergencies of international concern (PHEIC) by the World Health Organisation (WHO) [2, 3]. Other emerging infections such as the Middle East Respiratory Syndrome-related Coronavirus (MERS-CoV) and Lassa virus have caused costly regional epidemics with the potential to cause disease, death, and disruption of a magnitude equal to or greater than SARS-CoV-2 [4]. However, they have so far been prevented from causing a global pandemic.

Vaccines and diagnostics were critical in mitigating all declared PHEICs (except Zika, for which a licensed vaccination is unavailable) [5]. Non-pharmaceutical interventions such as movement restrictions, social distancing, school and workplace closures, contact tracing, and quarantine are essential emergency tools to mitigate pandemic impact, particularly for COVID-19 [6]. However, because of their temporary and costly nature, they cannot be used in isolation to end pandemics. Furthermore, their use is usually informed by diagnostics and complemented by vaccines.

As witnessed during the COVID-19 pandemic, vaccines and diagnostics can be inefficiently and inequitably used during pandemics [7]. Globally, equitable pandemic response has focused on initiatives like the Access to COVID-19 Tools Accelerator (ACT-A) [8] to facilitate the development of and financing for such technologies, with global agencies such as WHO providing broad recommendations on within-country deployment. In addition, a rigorous evidence-based framework is required to enable the efficient, equitable, and sustainable use of these technologies (and other countermeasures) in ways that reflect the values and needs of individual countries and population subgroups. A framework like this could also inform product development by directing developers to the most needed products across countries. This could particularly inform priorities for local production, as highlighted by Africa’s Centre for Disease Control and Prevention’s (ACDC) New Public Health Order [9].

The COVID-19 pandemic highlighted challenges in the availability and deployment of analytical tools to inform timely decision-making in all countries [10]. While several research institutions were rapidly able to provide epidemiological projections on disease spread and the impact of non-pharmaceutical interventions on outcomes such as hospitalizations and deaths under different scenarios [11], the integration of that information with related economic data proved more challenging. This was not a challenge solely for resource-constrained settings; higher-income countries also found it difficult to coordinate and integrate epidemiological and economic findings into coherent analyses [12].

A relevant and well-established framework for evidence-informed priority-setting is health technology assessment (HTA) [13]. With economic evaluation usually at its core, HTA provides both technical and procedural elements to evaluate the value for money of an existing or pipeline technology, such as a vaccine or diagnostic [14]. It applies multiple criteria, such as disease burden, intervention impact, cost-effectiveness, acceptability, feasibility, and equity, in a way that is seen as credible and legitimate. HTA is particularly important for low- and middle-income countries (LMICs), where the resource envelope to be divided for the health system is much smaller [15]. This is particularly acute during pandemics when countries face critical decisions in balancing pandemic investments with funding for essential endemic diseases. Even where investments are funded externally, they still require complementary domestic resources such as staffing. Many LMICs are in the process of institutionalizing HTA to inform resource allocation and support ambitions in achieving universal health coverage (UHC) [15].

Unfortunately, during an emergency (e.g., the COVID-19 pandemic), nascent local capacity in LMICs may not be able to generate relevant evidence rapidly and robustly enough to inform decision-making. While some evidence to inform HTA of both vaccines and diagnostics was generated during the COVID pandemic [16–19], it was limited and often came after critical decisions had been made. This was particularly the case among African countries.

HTA could be used to explore a number of pandemic/outbreak-related policy issues, including the evaluation of interventions to prepare for possible pandemics (stocking up antivirals, personal protective equipment, etc.); the review of interventions related to the prevention and control of pandemics (care and treatment in ICU, vaccines) as well as national programs on immunizations (NPIs). HTA could be used to identify relatively low-value interventions to minimize the adverse consequences of service displacement that can occur during pandemic/severe outbreak situations. However, there is a shortage of methods and procedural guidance on approaching HTA in the context of pandemics. Early HTA [20], which assesses technologies before their development, has gained increasing attention given its potential for guiding medical innovation development. It aims to help innovators understand the potential value of technologies, highlight key critical information gaps, and guide efficient research and development towards technologies that are most needed. It will likely be a beneficial approach in pandemic situations given the speed at which decisions need to be made.

There is a need to understand better how the experience of COVID-19 as well as other infectious diseases with pandemic potential has utilized and informed the application of HTA-like approaches to support prioritization in pandemic situations, focused on LMIC, and in particular African settings. The proposed review aims to identify stakeholders, pandemic preparedness and response (PPR) in Africa within the priority setting and resource allocation frame, including governance, coordination, and decision-making processes, incident management, strategies to support ongoing essential health services amid a pandemic, and organizational communication during a pandemic and community engagements, as it relates to supporting resource allocation choices. The review will also document best practices, challenges, capacity needs for HTA, and areas for improvement. This is important and can be recommended for African countries, especially those still trying to develop the use of HTA for decision-making in their countries and the findings of the review will be beneficial to their governments, their disease control agencies, and the various disease program officers.

The planned work, led by the University of Nigeria team, will leverage an existing collaboration based on work done on assessing the cost-effectiveness of COVID-19 vaccines [19], increased global interest and importance of pandemic preparedness, and the role of HTA in providing a valuable framework to address priority setting linked to infectious disease threats.

## Methodology

### Scoping review

The purpose of this protocol is to conduct a scoping review of the literature on pandemic preparedness, looking at literature from across the African continent. This is to inform a later situational assessment focused on how HTA-related capacities could be developed within existing institutional structures in Nigeria. The scoping review approach [21] was selected because it strives to provide an overview of the many types of evidence in the area of interest and identify the gaps that require further research. The review will be informed by recently published work on COVID-19 health system preparedness in Africa [22]. The Preferred Reporting Items for Systematic Reviews and Meta-Analyses Extension for Scoping Reviews (PRISMA-ScR) guidelines [23, 24] will be followed in reporting this review to map evidence on the topic and identify main concepts, theories, sources, and knowledge gaps.

The scoping review will adhere to the following procedures:

- 1. Determining the research questions
- 2. Identifying the relevant research articles
- 3. Selection of eligible articles
- 4. Analyzing the gathered data
- 5. Compiling and summarizing the results.

(i). Identifying the research questions

The main research questions are

- Who are the stakeholders involved in pandemic preparedness and response; What are their roles and contributions to emergency preparedness and response in Africa?
- What are the policies, strategies, action plans, strengthened systems, and operational readiness in place to ensure timely deployment and use of evidence to support priority setting following the identification of a potential epidemic or pandemic in Africa?
- What are the existing governance/coordination structures and decision-making processes for the control of pandemics in Africa?
- In terms of evidence evaluation and priority setting, what are the best practices and areas for improvement in pandemic preparedness and response in Africa?
- What is the national, and regional capacity to support data/evidence sharing within and between African countries and the international community?

### Inclusion criteria

In order for studies to be considered, they must satisfy the following requirements:

Published, preprint, or grey literature in the English language of full reviews, qualitative, quantitative, or mixed-methods design studies published between 2010–2024 that explore:

- Health system readiness/preparedness and Africa
- Responses to pandemic
- Priority setting and HTA

### Exclusion criteria

Studies with the following attributes will be omitted:

- Articles that do not include Africa or the African country experience
- Articles focused solely on the biological and clinical aspects of the disease
- Articles focused on building manufacturing capacity on the continent in relation to, for example, developing medical countermeasures

(ii). Identifying relevant studies

### Study sources

Peer-reviewed and grey literature sources will be used to identify relevant studies. Electronic databases will be used, for example, PubMed, Web of Science, Embase, Scopus, and Google Scholar lists of included peer-reviewed articles will be used as sources of peer-reviewed literature. These databases contain extensive abstracts and citations from a wide range of academic publications, including conference proceedings, books, and scientific journals. The databases also have a rich research output in the field of medicine and health sciences, as well as a flexible search engine for retrieving articles.

A comprehensive search will be conducted to locate both published and unpublished (grey) material on the specified topic, stakeholders, and components of pandemic preparedness and response (PPR) in Africa within the priority setting and resource allocation processes. The relevant, peer-reviewed publications and pertinent grey literature will be looked up in the electronic databases using focused web searches utilizing the key terms. When the full text of the articles is not available, then they will be excluded and added as reasons for exclusion. Study protocols will also not be included in the reviews.

### Search strategy

The search strategy will involve the identification of keywords from the research questions that comprehensively cover the topic of interest. The keywords will be tabulated, and their respective variations captured/specified. We will use keywords to capture all articles published on Health Technology Assessment (HTA) to support future pandemic preparedness. In addition, we will extend this search to identify relevant articles across Africa using additional keywords such as priority setting, evidence, and decision-making. Based on these keywords, search strings will be developed for scientific databases. The search strings for the scientific literature will be customized based on the requirements of each database considered in this study (Appendix 1). Table 1 shows the keywords and some variations of the keywords for the search.

**Table 1:** Keywords for the search

Keywords	Variation of the keywords
Health Technology Assessment	HTA, health technology assessment, economic evaluation, cost-effectiveness, cost-utility analysis
Priority setting	Priority setting, resource allocation, decision making
Africa	Africa, Nigeria, Algeria, Angola, Benin Republic, Botswana, Burkina Faso, Burundi, Cameroon, Cape Verde, CAR, Chad Republic, Comoros, Congo Brazzaville, DR Congo, Djibouti, Egypt, Equatorial Guinea, Eritrea, Ethiopia, Gabon, Gambia, Ghana, Guinea Conakry, Guinea Bissau, Ivory Coast, Kenya, Lesotho, Libya, Liberia, Madagascar, Mali, Malawi, Mauritius, Mauritania, Morocco, Mozambique, Niger Republic, Namibia, Sierra Leone, South Africa, Sudan, Senegal, Somalia, Rwanda, South Sudan, Swaziland, Sao Tome & Principe, Seychelles, Sub Saharan Africa, Tanzania, Togo, Tunisia, Uganda, Zambia, Zimbabwe
Pandemic	Epidemic, infection, infestation, outbreak, Emergence, Emergency, Epidemic, Crisis, Disaster, Ebola, COVID-19, SARS-CoV-2, swine flu, HINI, Polio, Zika, monkeypox, MERS-CoV, Lassa

*Generic search string:* see Appendix 2 for the generic search strings used to search for eligible studies.

The search method will be tested to see if the databases and keywords are adequate and sensitive enough to identify relevant articles. Based on the outcomes of the test searches, the search string will be modified to improve its suitability in collecting relevant literature. The electronic database search will be documented in a table.

(iii). Selection of eligible studies

The review will cover from 2010 to 2024. We chose 2010 as the beginning because going beyond this year is unlikely to provide relevant evidence for the present context. The eligible articles will be selected after screening the titles and abstracts for eligibility. The complete text will then be reviewed using the inclusion and exclusion criteria. The incorporation of papers that fulfill the requirement for eligibility will assist in addressing the research questions.

**Fig. 1:**
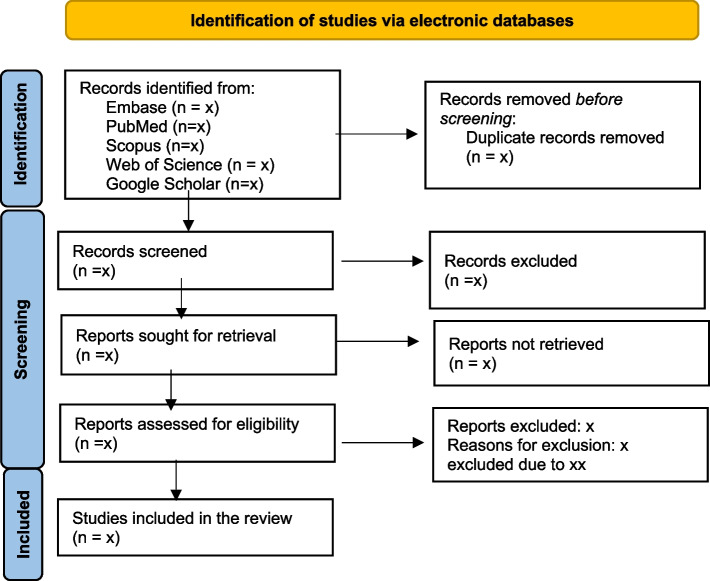
PRISMA flowchart. Adapted from [22]

Articles from our searches will be uploaded to the Covidence software, which will be used for removing duplicates, title and abstract screening, and full-text reading. A check for the number of duplicate articles Covidence removed will be done by clicking on the Import Studies button at the top of the review. After deduplication, articles will undergo a screening process for inclusion, and this will be conducted by a minimum of two independent researchers, who will evaluate the titles and abstracts. Full-text reading of articles and policy documents will also be done independently by the researchers to confirm inclusion in the review. Any disagreements on eligibility between researchers will be resolved by discussion or consensus. The researchers will make efforts to obtain the complete texts of the chosen publications, either by online searches or by reaching out to all the authors of the study if required. All suitable articles will be uploaded to the Covidence software, where any duplicates will be discovered and then eliminated. The Fig. 1 below shows the PRISMA flowchart for eligible studies that will be included in the review.

(iv) Data extraction

The data will be extracted with the use of a data extraction template which will be developed by the researchers. The template will be created in MS Excel and will include information from the article or policy document such as author names, year of publication, title, goal, and relevant content that answers the research questions. The data that will be extracted will consist of the following fields (see Appendix 3).

(v) Collating, summarizing, and reporting the results

We will use a thematic content analysis using narrative descriptions of the extracted data, around the following: country of study, types of stakeholders involved in pandemic preparedness and response; Their duties and contributions in the context of emergency preparedness and response. The various policies, strategies, and action plans aimed at ensuring timely deployment and use of evidence to support priority setting following a potential pandemic in Africa will be covered. Governance and coordination structures, as well as decision-making processes for pandemic control in Africa, including the use of HTA and other evidence-based processes, will be reported. Best practices, constraints, and opportunities for improvement will be identified. Appendix 4 shows the template for the narrative report. The data from quantitative studies will be collated and reported as narratives of the findings. Their tables will not be included in this study.

The review findings will be discussed with respect to the research questions and the general goal of the study. Gap analysis will identify certain places, such as countries in SSA, where there are significant deficiencies or shortcomings, with their PPR plans and strategies, capacity needs for PPR, and HTA for priority setting.

The situational analysis will seek to draw on the literature review to develop a rapid assessment of the institutions and institutional processes that could be involved in any future pandemic and outbreak management scenario. This assessment would look at areas of strength and weakness, especially with respect to priority setting and the collection and use of evidence, and identify opportunities for capacity building. The main area to explore is how HTA-like systems could be used to support policy-makers in pandemic scenarios. The situational analysis will be developed based on the recently created International Decision Support Initiative (iDSI) guidance and reporting template [25], and also draw on earlier work by Uzochukwu and colleagues, 2019 [13].

## Discussion

The objective of the proposed review is to identify and gain an understanding of the impact of COVID-19 and other global public health emergencies on individuals, such as influenza H1N1, Ebola, Polio, Zika, monkeypox, MERS-CoV and Lassa virus have involved and/or informed the application of HTA-like approaches to support prioritization in pandemic situations, focused on LMIC, and in particular African settings. It will highlight the existing institutional relationships and processes associated with pandemic preparedness relevant to an African setting, focusing on priority setting and resource allocation. A rapid situational analysis of the potential HTA and related evidence-driven approaches to support pandemic preparedness, with a focus on relevant institutions (and institutional processes) involved in future pandemic and possible outbreak management scenarios will later be developed.

The review will make recommendations on likely improvements to current approaches to evidence use in priority settings during pandemics and emergencies in the African region. The review will also identify capacity-strengthening needs that should be addressed. This review has the potential to generate more awareness of the field of priority setting within the context of pandemic preparedness and response. It will document some of the best practices and reveal gaps in pandemic preparedness and response. These will enable African countries’ governments and program officers to learn from their experiences and those of other countries. It will also inspire strategic organization of future epidemic/pandemic responses.

### Dissemination and ethics

This study is a retrospective analysis of publicly accessible secondary data. Therefore, obtaining ethical clearance is not required. The options for dissemination of the results will include stakeholder workshops and through a peer-reviewed publication, blogs, and Twitter.

## Data Availability

All the data that is generated and analyzed will be incorporated into the published scoping review paper.

## Appendix 1: PubMed search strategy

**Table:**

Query	Search details
(((((((("Health technology assessment") OR ((Health Technology Assessment) OR (Health Technology Assessment))) OR (HTA)) OR (economic evaluation)) OR (Cost effectiveness analysis)) OR (Cost utility analysis)) AND ((((Priority setting) OR ("priority setting")) OR (resource allocation)) OR (Decision making))) AND (Nigeria OR Ethiopia OR Egypt OR Democratic Republic of Congo OR DRC OR Tanzania OR South Africa OR Kenya OR Uganda OR Algeria OR Sudan OR Morocco OR Angola OR Mozambique OR Ghana OR Madagascar OR Cameroon OR Ivory Coast OR Niger OR Burkina Faso OR Mali OR Malawi OR Zambia OR Senegal OR Chad OR Somalia OR Zimbabwe Or Guinea OR Rwanda OR Benin OR Burundi OR South Sudan OR Tunisia OR Togo OR Serria Leone OR Libya OR Congo OR Liberia OR Central African Republic OR Namibia OR Eritrea OR Mauritania OR Gambia OR Botswana OR Gabon OR Lesotho OR Guinea Bissau OR Equatorial Guinea OR Mauritius OR Eswatini OR Swaziland OR Djibouti OR Comoros OR Cabo Verde OR Sao Tome & Principe OR Seychelles OR Sub Saharan Africa OR Africa)) AND (Pandemic OR Outbreak OR Emergence OR Emergency OR Epidemic OR Crisis OR Disaster OR Ebola OR COVID-19 OR SARS-CoV-2)	("Health technology assessment"[All Fields] OR ("technology assessment, biomedical"[MeSH Terms] OR ("technology"[All Fields] AND "assessment"[All Fields] AND "biomedical"[All Fields]) OR "biomedical technology assessment"[All Fields] OR ("health"[All Fields] AND "technology"[All Fields] AND "assessment"[All Fields]) OR "Health technology assessment"[All Fields] OR ("technology assessment, biomedical"[MeSH Terms] OR ("technology"[All Fields] AND "assessment"[All Fields] AND "biomedical"[All Fields]) OR "biomedical technology assessment"[All Fields] OR ("health"[All Fields] AND "technology"[All Fields] AND "assessment"[All Fields]) OR "Health technology assessment"[All Fields])) OR ("health technol assess"[Journal] OR "hta"[All Fields]) OR ("cost benefit analysis"[MeSH Terms] OR OR Cost utility analysis"[All Fields])) AND ((("priorities"[All Fields] OR "priority"[All Fields]) AND ("setting"[All Fields] OR "setting s"[All Fields] OR "settings"[All Fields])) OR "priority setting"[All Fields] OR ("resource allocation"[MeSH Terms] OR ("resource"[All Fields] AND "allocation"[All Fields]) OR "resource allocation"[All Fields]) OR ("decision making"[MeSH Terms] OR ("decision"[All Fields] AND "making"[All Fields]) OR "decision making"[All Fields])) AND ((("Nigeria"[MeSH Terms] OR "Nigeria"[All Fields] OR "Nigeria OR "Ethiopia"[All Fields] OR "Ethiopia "[All Fields]) OR ("egypt"[MeSH Terms] OR "egypt"[All Fields] OR "Egypt "[All Fields]) OR (("Democrat"[All Fields] OR "Democratic"[All Fields] OR "Democratically"[All Fields] OR "Democratization"[All Fields] OR "Democratize"[All Fields] OR "Democratized"[All Fields] OR "Democratizing"[All Fields] OR "Democrats"[All Fields]) AND ("Republic"[All Fields] OR "Republic s"[All Fields] OR "Republics"[All Fields]) AND ("Congo"[MeSH Terms] OR "Congo"[All Fields])) OR "DRC" OR "Tanzania s"[All Fields]) OR ("south africa"[MeSH Terms] OR ("south"[All Fields] AND "africa"[All Fields]) OR "south africa"[All Fields]) OR "Kenya"[All Fields] OR "Kenya"[All Fields]) OR ("uganda"[MeSH Terms] OR "uganda"[All Fields] OR "uganda "[All Fields]) OR ("algeria"[MeSH Terms] OR "algeria"[All Fields]) OR "Sudans"[All Fields] OR "Sudan"[All Fields]) OR ("Morocco"[MeSH Terms] OR "Angola"[All Fields]) OR ("Mozambique"[MeSH Terms] OR "Mozambique"[All Fields] OR "Mozambique "[All Fields]) OR "Ghana"[All Fields]) OR ("Madagascar"[MeSH Terms] OR "Madagascar"[All Fields] OR "Madagascar "[All Fields]) OR ("Cameroon"[MeSH Terms] OR All Fields] OR "Cameroons"[All Fields]) OR ("cote d ivoire"[MeSH Terms] OR ("cote"[All Fields] AND "d ivoire"[All Fields]) OR "cote d ivoire"[All Fields] OR ("Ivory"[All Fields] AND "Coast"[All Fields]) OR "Ivory Coast"[All Fields]) OR ("niger"[MeSH Terms] OR "Niger"[All Fields]) OR ("Burkina"[All Fields] AND "faso"[All Fields]) OR "Burkina faso"[All Fields]) OR ("Mali"[MeSH Terms] OR "Mali"[All Fields]) OR ("Malawi"[MeSH Terms] OR "Malawi"[All Fields] OR "Malawis"[All Fields]) OR "Zambias"[All Fields]) OR ("Senegal"[MeSH Terms] OR "senegal"[All Fields] OR "Senegals"[All Fields]) OR ("Chad"[MeSH Terms]) OR "Somalia"[All Fields] OR "somalia s"[All Fields]) OR ("zimbabwe"[MeSH Terms] OR "zimbabwe"[All Fields] OR "zimbabwe"[All Fields]) AND "Or"[All Fields] AND ("Guinea"[MeSH Terms] OR "guinea"[All Fields] OR "Guinea"[All Fields] OR "Guineas"[All Fields])) OR ("Rwanda"[MeSH Terms] OR "Rwanda"[All Fields] OR "Rwandas"[All Fields]) OR ("Benin Republic"[MeSH Terms] OR "benin republic"[All Fields] OR "benins"[All Fields]) OR "Burundi"[All Fields]) OR "south sudan"[All Fields]) OR ("tunisia"[MeSH Terms] OR "Tunisia"[All Fields]) OR "Togo"[All Fields]) OR ("Serria"[All Fields] AND ("leone"[All Fields] OR "leones"[All Fields])) OR ("libya"[MeSH Terms] OR "libya"[All Fields]) OR ("congo"[MeSH Terms] OR "congo"[All Fields]) OR ("liberia"[MeSH Terms] OR "liberia"[All Fields]OR "central african republic"[All Fields]) OR ("namibia"[MeSH Terms] OR "namibia"[All Fields] OR "namibia s"[All Fields]) OR ("eritrea"[MeSH Terms] OR "eritrea"[All Fields]) OR ("mauritania"[MeSH Terms] OR "mauritania"[All Fields]) OR ("gambia"[MeSH Terms] OR "gambia"[All Fields] OR "gambia s"[All Fields]) OR "botswana s"[All Fields]) OR ("gabon"[MeSH Terms] OR "gabon"[All Fields]) OR ("lesotho"[MeSH Terms] OR "lesotho"[All Fields]) OR ("guinea bissau"[MeSH Terms] OR "guinea bissau"[All Fields]) OR ("equatorial"[All Fields] AND "guinea"[All Fields]) OR "equatorial guinea"[All Fields]) OR ("mauritius"[MeSH Terms] OR "mauritius"[All Fields]) OR ("eswatini"[MeSH Terms] OR "eswatini"[All Fields]) OR ("eswatini"[MeSH Terms] OR "eswatini"[All Fields] OR "swaziland"[All Fields]) OR "djibouti"[All Fields]) OR "comoros"[All Fields] OR ("cabo"[All Fields] AND "verde"[All Fields]) OR "cabo verde"[All Fields]) OR ("Sao"[All Fields] AND "Tome"[All Fields])) AND ("principe"[All Fields] OR ("seychelles"[MeSH Terms] OR "seychelles"[All Fields]) OR ("africa south of the sahara"[MeSH Terms] OR "pandemicity"[All Fields] OR "pandemics"[MeSH Terms] OR "pandemics"[All Fields] OR "pandemic"[All Fields] OR ("disease outbreaks"[MeSH Terms] OR ("disease"[All Fields] AND "outbreaks"[All Fields]) OR "disease outbreaks"[All Fields] OR "outbreak"[All Fields] OR "epidemiology"[MeSH Subheading] OR "outbreaks"[All Fields] OR ("emerge"[All Fields] OR "emerged"[All Fields] OR "emergence"[All Fields] OR "emergences"[All Fields] OR "emergencies"[MeSH Terms] OR "emergencies"[All Fields] OR "emergency"[All Fields] OR "emergent"[All Fields] OR "emerges"[All Fields] OR "emerging"[All Fields]) OR ("emerge"[All Fields] OR "emerged"[All Fields] OR "emergence"[All Fields] OR "emergences"[All Fields] OR "emergencies"[MeSH Terms] OR "emergencies"[All Fields] OR "emergency"[All Fields] OR ("epidemic s"[All Fields] OR "epidemical"[All Fields] OR "epidemically"[All Fields] OR "epidemicity"[All Fields] OR "epidemics"[MeSH Terms] OR "epidemics"[All Fields] OR "epidemic"[All Fields] OR "epidemiology"[MeSH Subheading] OR "epidemiology"[All Fields]) OR ("crisis"[Journal] OR "crisis"[All Fields]) OR ("disasters"[All Fields] OR "disasters"[MeSH Terms] OR "disasters"[All Fields] OR "disaster"[All Fields]) OR ("hemorrhagic fever, ebola"[MeSH Terms] OR ("hemorrhagic"[All Fields] AND "fever"[All Fields] AND "ebola"[All Fields]) OR "ebola hemorrhagic fever"[All Fields] OR "ebola"[All Fields] OR "ebolavirus"[MeSH Terms] OR "ebolavirus"[All Fields]) OR ("covid 19"[All Fields] OR "covid 19"[MeSH Terms]
Pandemic OR Outbreak OR Emergence OR Emergency OR Epidemic OR Crisis OR Disaster OR Ebola OR COVID-19 OR SARS-CoV-2	"pandemic s"[All Fields] OR "pandemically"[All Fields] OR "pandemicity"[All Fields] OR "pandemics"[MeSH Terms] OR "emerging"[All Fields]) OR ("epidemic s"[All Fields] OR "epidemical"[All Fields] OR "epidemically"[All Fields] OR "epidemicity"[All Fields] OR "epidemics"[MeSH Terms] OR "epidemics"[All Fields] OR "epidemic"[All Fields] OR "epidemiology"[MeSH Subheading] OR "epidemiology"[All Fields]) OR ("crisis"[Journal] OR "crisis"[All Fields]) OR ("disaster s"[All Fields] OR "disasters"[MeSH Terms] OR "disasters"[All Fields] OR "disaster"[All Fields]) OR ("hemorrhagic fever, ebola"[MeSH Terms] OR ("hemorrhagic"[All Fields] AND "fever"[All Fields] AND "ebola"[All Fields]) OR "ebola hemorrhagic fever"[All Fields] OR "ebola"[All Fields] OR "ebolavirus"[MeSH Terms] OR "ebolavirus"[All Fields]) OR ("covid 19"[All Fields] OR "covid 19"[MeSH Terms] OR "coronavirus"[All Fields] OR "cov"[All Fields])
Nigeria OR Ethiopia OR Egypt OR Democratic Republic of Congo OR DRC OR Tanzania OR South Africa OR Kenya OR Uganda OR Algeria OR Sudan OR Morocco OR Angola OR Mozambique OR Ghana OR Madagascar OR Cameroon OR Ivory Coast OR Niger OR Burkina Faso OR Mali OR Malawi OR Zambia OR Senegal OR Chad OR Somalia OR Zimbabwe Or Guinea OR Rwanda OR Benin OR Burundi OR South Sudan OR Tunisia OR Togo OR Serria Leone OR Libya OR Congo OR Liberia OR Central African Republic OR Namibia OR Eritrea OR Mauritania OR Gambia OR Botswana OR Gabon OR Lesotho OR Guinea Bissau OR Equatorial Guinea OR Mauritius OR Eswatini OR Swaziland OR Djibouti OR Comoros OR Cabo Verde OR Sao Tome & Principe OR Seychelles OR Sub Saharan Africa OR Africa	(("nigeria"[MeSH Terms] OR "nigeria"[All Fields] OR "nigeria s"[All Fields] OR ("ethiopia"[MeSH Terms OR " OR "egypt s"[All Fields]) OR (("democrat"[All Fields] OR "democratic"[All Fields] OR "democratize"[All Fields] OR "democratized"[All Fields] OR "democratizing"[All Fields] OR "democrats"[All Fields]) AND ("republic"[All Fields] OR " OR "DRC"[All Fields] OR ("tanzania"[MeSH Terms] OR "tanzania"[All Fields] OR "tanzania s"[All Fields]) OR "south africa"[All Fields]) OR ("kenya"[MeSH Terms] OR "kenya"[All Fields] OR ("uganda"[MeSH Terms] OR "uganda"[All Fields] OR ("algeria"[MeSH Terms] OR "algeria"[All Fields]) OR ("sudan"[MeSH Terms] OR "sudan"[All Fields] OR "sudan s"[All Fields]) OR ("morocco"[MeSH Terms] OR "morocco"[All Fields]) OR ("angola"[MeSH Terms] OR "angola"[All Fields] OR "angolas"[All Fields]) OR ("mozambique"[MeSH Terms] OR "mozambique"[All Fields] OR ("ghana"[MeSH Terms] OR "ghana"[All Fields] OR "ghana s"[All Fields]) OR ("madagascar"[MeSH Terms] OR "madagascar"[All Fields] OR ("cameroon"[MeSH Terms] OR "cameroon"[All Fields] OR "cameroons"[All Fields] OR "cameroon s"[All Fields]) OR ("cote d ivoire"[MeSH Terms] OR ("cote"[All Fields] AND "d ivoire"[All Fields]) OR "niger"[All Fields]) OR ("burkina faso"[MeSH Terms] OR ("burkina"[All Fields] AND "faso"[All Fields]) OR ("mali"[MeSH Terms] OR "mali"[All Fields]) OR ("malawi"[MeSH Terms] OR "malawi s"[All Fields]) OR ("zambia"[MeSH Terms] OR "zambia"[All Fields] OR ("senegal"[MeSH Terms] OR "senegal"[All Fields] OR "senegal s"[All Fields]) OR ("chad"[MeSH Terms] OR "chad"[All Fields]) OR ("somalia"[MeSH Terms] OR "somalia s"[All Fields]) OR (("zimbabwe"[MeSH Terms] OR "zimbabwe"[All Fields] OR "zimbabwe s"[All Fields]) AND "Or"[All Fields] AND ("guinea"[MeSH Terms] OR "guinea"[All Fields] OR ("rwanda"[MeSH Terms] OR "rwanda"[All Fields] OR "rwanda s"[All Fields]) OR ("benin"[MeSH Terms] OR "benin"[All Fields] OR "benin s"[All Fields]) OR ("burundi"[MeSH Terms] OR "burundi"[All Fields]) OR ("south sudan"[MeSH Terms] OR ("south"[All Fields] AND "sudan"[All Fields]) OR "south sudan"[All Fields]) OR ("tunisia"[MeSH Terms] OR "tunisia"[All Fields]) OR ("togo"[MeSH Terms] OR "togo"[All Fields]) OR ("Serria"[All Fields] AND ("leone"[All Fields] OR "leone s"[All Fields] OR "leones"[All Fields])) OR ("libya"[MeSH Terms] OR "libya"[All Fields]) OR ("congo"[MeSH Terms] OR "congo"[All Fields]) OR ("liberia"[MeSH Terms] OR "liberia"[All Fields] OR "liberia s"[All Fields]) OR ("central african republic"[MeSH Terms] OR ("central"[All Fields] AND "african"[All Fields] AND "republic"[All Fields]) OR "central african republic"[All Fields]) OR ("namibia"[MeSH Terms] OR "namibia"[All Fields] OR "namibia s"[All Fields]) OR ("eritrea"[MeSH Terms] OR "eritrea"[All Fields]) OR ("mauritania"[MeSH Terms] OR "mauritania"[All Fields]) OR ("gambia"[MeSH Terms] OR "gambia"[All Fields] OR "gambia s"[All Fields]) OR ("botswana"[MeSH Terms] OR "botswana"[All Fields] OR "botswana s"[All Fields]) OR ("gabon"[MeSH Terms] OR "gabon"[All Fields]) OR ("lesotho"[MeSH Terms] OR "lesotho"[All Fields]) OR ("guinea bissau"[MeSH Terms] OR "guinea bissau"[All Fields] OR ("guinea"[All Fields] AND "bissau"[All Fields]) OR "guinea bissau"[All Fields]) OR ("equatorial guinea"[MeSH Terms] OR ("equatorial"[All Fields] AND "guinea"[All Fields]) OR "equatorial guinea"[All Fields]) OR ("mauritius"[MeSH Terms] OR "mauritius"[All Fields]) OR ("eswatini"[MeSH Terms] OR "eswatini"[All Fields]) OR ("eswatini"[MeSH Terms] OR "eswatini"[All Fields] OR "swaziland"[All Fields]) OR ("djibouti"[MeSH Terms] OR "djibouti"[All Fields]) OR ("comoros"[MeSH Terms] OR "comoros"[All Fields] OR "comoro"[All Fields]) OR ("cabo verde"[MeSH Terms] OR ("cabo"[All Fields] AND "verde"[All Fields]) OR "cabo verde"[All Fields]) OR ("Sao"[All Fields] AND "Tome"[All Fields])) AND ("principe"[All Fields] OR "principes"[All Fields])) OR ("seychelles"[MeSH Terms] OR "seychelles"[All Fields]) OR ("africa south of the sahara"[MeSH Terms] OR "africas"[All Fields])
(((Priority setting) OR ("priority setting")) OR (resource allocation)) OR (Decision making)	(("priorities"[All Fields] OR "priority"[All Fields]) AND ("setting"[All Fields] OR "setting s"[All Fields] OR "settings"[All Fields])) OR "priority setting"[All Fields] OR ("resource allocation"[MeSH Terms] OR ("resource"[All Fields] AND "allocation"[All Fields]) OR "resource allocation"[All Fields]) OR ("decision making"[MeSH Terms] OR ("decision"[All Fields] AND "making"[All Fields]) OR "decision making"[All Fields])
Decision making	"decision making"[MeSH Terms] OR ("decision"[All Fields] AND "making"[All Fields]) OR "decision making"[All Fields]
((Priority setting) OR ("priority setting")) OR (resource allocation)	(("priorities"[All Fields] OR "priority"[All Fields]) AND ("setting"[All Fields] OR "setting s"[All Fields] OR "settings"[All Fields])) OR "priority setting"[All Fields] OR ("resource allocation"[MeSH Terms] OR ("resource"[All Fields] AND "allocation"[All Fields]) OR "resource allocation"[All Fields])
resource allocation	"resource allocation"[MeSH Terms] OR ("resource"[All Fields] AND "allocation"[All Fields]) OR "resource allocation"[All Fields]
"priority setting"	"priority setting"[All Fields]
Priority setting	("priorities"[All Fields] OR "priority"[All Fields]) AND ("setting"[All Fields] OR "setting s"[All Fields] OR "settings"[All Fields])
((((("Health technology assessment") OR ((Health Technology Assessment) OR (Health Technology Assessment))) OR (HTA)) OR (economic evaluation)) OR (Cost effectiveness analysis)) OR (Cost utility analysis)	"Health technology assessment"[All Fields] OR ("technology assessment, biomedical"[MeSH Terms] OR ("technology"[All Fields] AND "assessment"[All Fields] AND "biomedical"[All Fields]) OR "biomedical technology assessment"[All Fields] OR ("health"[All Fields] AND "technology"[All Fields] AND "assessment"[All Fields]) OR "Health technology assessment"[All Fields] OR ("technology assessment, biomedical"[MeSH Terms] OR ("technology"[All Fields] AND "assessment"[All Fields] AND "biomedical"[All Fields]) OR "biomedical technology assessment"[All Fields] OR ("health"[All Fields] AND "technology"[All Fields] AND "assessment"[All Fields]) OR "Health technology assessment"[All Fields])) OR ("cost benefit analysis"[MeSH Terms] OR ("cost benefit"[All Fields] AND "analysis"[All Fields]) OR ("cost effectiveness analysis"[MeSH Terms] OR ("cost effectiveness"[All Fields] AND "analysis"[All Fields]) OR ("cost"[All Fields] AND "utility"[All Fields] AND "analysis"[All Fields]) OR "cost utility analysis"[All Fields])
Cost utility analysis	"Cost utility"[All Fields] AND "analysis"[All Fields]) OR "cost utility analysis"[All Fields]
Cost effectiveness analysis	"cost effectiveness analysis"[MeSH Terms] OR ("cost effectiveness"[All Fields] AND "analysis"[All Fields]) OR "cost effectiveness analysis"[All Fields] OR ("cost"[All Fields] AND "effectiveness"[All Fields] AND "analysis"[All Fields]) OR "cost effectiveness analysis"[All Fields]
economic evaluation	"cost benefit analysis"[All Fields] OR ("economic"[All Fields] AND "evaluation"[All Fields]) OR "economic evaluation"[All Fields]
HTA	"health technol assess"[Journal] OR "hta"[All Fields]
(Health Technology Assessment) AND (Health Technology Assessment)	("technology assessment, biomedical"[MeSH Terms] OR ("technology"[All Fields] AND "assessment"[All Fields] AND "biomedical"[All Fields]) OR "biomedical technology assessment"[All Fields] OR ("health"[All Fields] AND "technology"[All Fields] AND "assessment"[All Fields]) OR "health technology assessment"[All Fields]) AND ("technology assessment, biomedical"[MeSH Terms] OR ("technology"[All Fields] AND "assessment"[All Fields] AND "biomedical"[All Fields]) OR "biomedical technology assessment"[All Fields] OR ("health"[All Fields] AND "technology"[All Fields] AND "assessment"[All Fields]) OR "health technology assessment"[All Fields])
"Health technology assessment"	"Health technology assessment"[All Fields]

## Appendix 2: The generic search string

The generic search string will be: (((((((("Health technology assessment") OR ((Health Technology Assessment) OR (Health Technology Assessment))) OR (HTA)) OR (economic evaluation)) OR (Cost effectiveness analysis)) OR (Cost utility analysis)) AND ((((Priority setting) OR ("priority setting")) OR (resource allocation)) OR (Decision making))) AND (Nigeria OR Ethiopia OR Egypt OR Democratic Republic of Congo OR DRC OR South Africa OR Tanzania OR Kenya OR Uganda OR Algeria OR Sudan OR Morocco OR Angola OR Mozambique OR Ghana OR Cameroon OR Madagascar OR Ivory Coast OR Niger OR Burkina Faso OR Mali OR Malawi OR Senegal OR Chad OR Zambia OR Somalia OR Zimbabwe Or Guinea OR Rwanda OR Benin OR Burundi OR South Sudan OR Tunisia OR Togo OR Sierra Leone OR Libya OR Congo OR Liberia OR Central African Republic OR Namibia OR Eritrea OR Mauritania OR Gambia OR Botswana OR Gabon OR Lesotho OR Guinea Bissau OR Equatorial Guinea OR Mauritius OR Eswatini OR Swaziland OR Djibouti OR Comoros OR Cabo Verde OR Sao Tome & Principe OR Seychelles OR Sub Saharan Africa OR Africa)) AND (Pandemic OR Outbreak OR Emergence OR Emergency OR Epidemic OR Crisis OR Disaster OR Ebola OR COVID-19 OR SARS-CoV-2).

## Appendix 3: Data charting form

**Table:**

Source of document (eg PubMed, Google scholar, Organizational website, or Library/unpublished)	
Full citation of document (Note: only documents within 2010–2022)	Name of authors, title of the article, year of publication, page numbers/web address
Location covered in the study (country)	
Is the article national or regional (state the region if regional)	
Type of document (e.g., original article, documentary, media, blog)	
Objectives/purpose of study, paper, report etc	
Study design (quantitative, qualitative, mixed methods)	
Sampling method	
Data collection method	
Data Analysis	
Main findings related to objectives (Obj) 1–5	Obj 1: PPR activities in areas such as disease surveillance, laboratory services, health workforce, essential services continuation, emergency communication and management, and community engagement Obj 2: To support priority setting following identification of a potential pandemic, document identified policies to ensure timely deployment and use of evidence, strategies to ensure timely deployment and use of evidence, action plans to ensure timely deployment and use of evidence and strengthened systems and operational readiness to ensure timely deployment and use of evidence Obj 3: Document the governance and coordination structures identified and the decision-making processes for the control of pandemics in Africa including the use of Health Technology Assessment or similar evidence driven processes Obj 4: Document best practices and strengths in PPR in Africa, gaps in Knowledge with respect to specific document reviewed, and constraints and areas for improvement in PPR with respect to evidence evaluation and priority setting Obj 5: Document national, and regional capacity to support data sharing, regulatory harmonization, coordinated development and procurement, distribution and deployment of countermeasures and essential medical supplies
Most relevant findings	
Best practices, strengths, constraints and areas for improvement in PPR in Africa	
Gaps in Knowledge (with respect to specific document reviewed)	
Capacity needs identified	
Policy implications	
Remarks/comments	

## Appendix 4: Template for narrative report

**Table:**

Background characteristics	
**Name** *(Family name and given first name)*** of reviewer**	
**Date of review** *(dd/mm/yyyy)*	
**Complete reference of documents or databases** *(Harvard/APA/Chicago 16th Author-Date style)*	
**Source of information** *(e.g., Ministry of Health; PATHS 2; WHO website)*	
**Remarks**	
**Objectives**	**Narrative** (The following specific information points should be elicited to complement the findings for specific objectives.) Cite all sources of information
1.Document the various stakeholders involved in pandemic preparedness and response; their roles and contributions to emergency preparedness and response. (Please add the frameworks used where applicable and categorize the stakeholders)	*Document pandemic preparedness and response activities in the following areas* i. Disease surveillance and early warning signs ii. Laboratory systems iii. Health workforce iv. Essential services continuation v. Risk communication and management vi. Community engagement
2.Document established policies, strategies, action plans, strengthened systems and operational readiness to ensure timely deployment and use of evidence to support priority setting following identification of a potential pandemic in Africa	*Document the following:* i. Established policies to ensure timely deployment and use of evidence to support priority setting following identification of a potential pandemic ii. Strategies to ensure timely deployment and use of evidence to support priority setting following identification of a potential pandemic iii. Action plans to ensure timely deployment and use of evidence to support priority setting following identification of a potential pandemic iv. Strengthened systems and operational readiness to ensure timely deployment and use of evidence to support priority setting following identification of a potential pandemic
3.Document the governance and coordination structures identified, as well as decision making processes for the control of pandemics in Africa including the use of HTA or similar evidence driven processes	*Document the following:* i. The governance and coordination structures identified ii. The decision-making processes for the control of pandemics in Africa including the use of HTA or similar evidence driven processes
4. Document best practices, strengths, constraints and areas for improvement in pandemic preparedness and response with respect to evidence evaluation and priority setting, e.g., the generation of relevant local evidence, linked to improved capacity in for example, surveillance	*Document the following:* i. Best practices in pandemic preparedness and response with respect to evidence evaluation and priority setting ii. Strengths in pandemic preparedness and response with respect to evidence evaluation and priority setting iii. Constraints and areas for improvement in pandemic preparedness and response with respect to evidence evaluation and priority setting
5. Document national, regional, and global capacity, to support data sharing, and capacity for coordinated assessment of the effectiveness and cost of potential countermeasures	*Document the following:* i a. National capacity to support data sharing b. Regional capacity to support data sharing c. Global capacity to support data sharing ii a. National capacity to support regulatory harmonization, and capacity for coordinated development b. Regional capacity to support regulatory harmonization, and capacity for coordinated development c. Global capacity to support regulatory harmonization, and capacity for coordinated development iii a. National capacity to support regulatory harmonization b. Regional capacity to support regulatory harmonization c. Global capacity to support regulatory harmonization iv a. National capacity for procurement, distribution and deployment of countermeasures and essential medical supplies b. Regional capacity for procurement, distribution and deployment of countermeasures and essential medical supplies c. Global capacity for procurement, distribution and deployment of countermeasures and essential medical supplies

## Abbreviations

ACT-A: Access to COVID-19 Tools Accelerator
CDC: Centre for Disease Control and Prevention
HTA: Health Technology Assessment
ICU: Intensive care unit
iDSI: International Decision Support Initiative
LMIC: Low- and middle-income countries
MERS-CoV: Middle East Respiratory Syndrome -related Coronavirus
PHEIC: Public Health Emergency of International Concern
PPR: Pandemic Preparedness and Response
PRISMA-ScR: Preferred Reporting Items for Systematic Reviews and Meta-Analyses Extension for Scoping Reviews
UHC: Universal Health Coverage
WHO: World Health Organization
